# Development of a peptide-based vaccine using a T cell epitope derived from SARS-CoV-2

**DOI:** 10.1016/j.isci.2025.112542

**Published:** 2025-04-28

**Authors:** Satoshi Baba, Hiroki Hayashi, Shota Yoshida, Nanxiang Yin, Munehisa Shimamura, Ryuichi Morishita, Hiromi Rakugi, Hironori Nakagami, Koichi Yamamoto

**Affiliations:** 1 Department of Health Development and Medicine, Osaka University, Suita, Osaka, Japan; 2 Department of Geriatric Medicine, Osaka University, Suita, Osaka, Japan; 3 Department of Gene & Stem Cell Regenerative Therapy, Osaka University, Suita, Osaka, Japan; 4 Department of Neurology, Osaka University, Suita, Osaka, Japan; 5 Department of Clinical Gene Therapy, Osaka University, Suita, Osaka, Japan; 6 Osaka Rosai Hospital, Sakai, Osaka, Japan; 7 Center for Infectious Disease Education and Research (CiDER), Osaka University, Suita, Osaka, Japan

**Keywords:** Virology, Immunology

## Abstract

Follicular helper T (Tfh) cells are a subset of CD4^+^ T cells that help B cells to produce high-affinity antibodies. Efficient Tfh cell induction by vaccines is critical for protective efficacy against diseases. A Tfh epitope, identified from the SARS-CoV-2 spike protein, has been shown to bind to the corresponding HLA and activates Tfh cells. Here, we assessed the efficacy of a peptide vaccine conjugated with SARS-CoV-2 Tfh epitope against experimental hypertension in a mouse model. The Tfh-angiotensin II (Tfh-Ang II) vaccine activated Tfh and germinal center B cells and induced antibodies against Ang II, thereby suppressing hypertension. However, Ang II-specific autoreactive T cells were not induced. Interestingly, Tfh-Ang II-induced antibody production was enhanced by SARS-CoV-2 spike priming. Moreover, human peripheral blood mononuclear cells from individuals vaccinated with COVID-19 mRNA vaccine were activated by Tfh epitope. Collectively, the SARS-CoV-2 spike-derived universal Tfh epitope may be effective for peptide-based vaccine development.

## Highlights

• Peptide-based Ang II vaccine was generated with SARS-CoV-2 derived Tfh epitope
• Tfh epitope-Ang II vaccine upregulated Tfh cells and GC B cells in mice
• Tfh epitope-Ang II vaccine induced anti-Ang II antibody and antihypertensive effect
• Priming by SARS-CoV-2 S protein enhanced the effect of Tfh epitope-Ang II vaccine

## Graphical abstract

**Figure:**
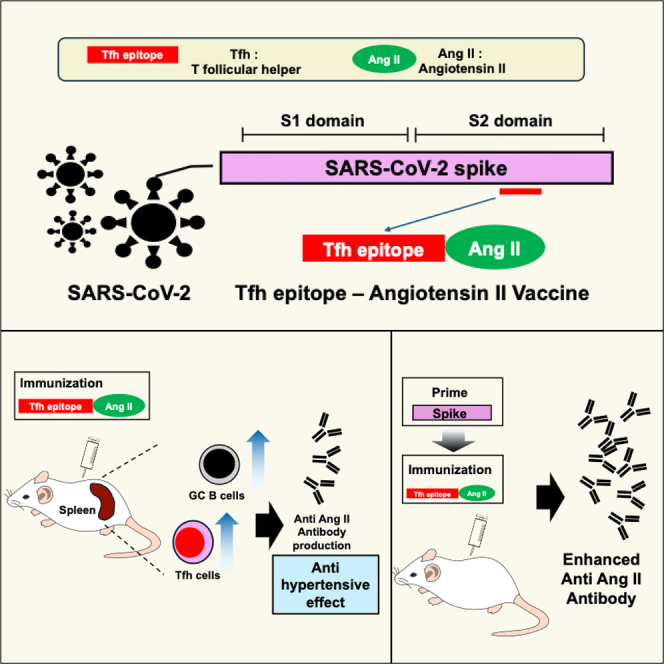

## Introduction

Vaccines are used to prevent infectious diseases, such as viral infections, and treat chronic diseases, such as hypertension and Alzheimer’s disease.^1^^,^^2^^,^^3^^,^^4^ Vaccine therapy against endogenous proteins has received especially a great deal of attention for various purposes, such as improving polypharmacy and adherence to internal medication.^4^ Peptide-based vaccines consist of a carrier protein containing T cell epitopes that activate CD4^+^ helper T cells, and B cell epitopes, which serve as the antigen.^3^ Following immunization with peptide-based vaccines, a few naive T cells differentiate into follicular helper T cells (Tfh cells), a subset of CD4^+^ T cells that help B cells produce high-affinity antibodies in the presence of IL-4, IL-6, and IL-21.^4^^,^^5^^,^^6^

Tfh cells have been identified as T cells that express the master regulator transcription factor Bcl6 and the chemokine receptor CXCR5.^7^^,^^8^^,^^9^ Tfh cells regulate the germinal center response, which regulates the production of germinal center-derived high-affinity antibodies, memory B cells, and long-lived plasma cells, constituting the basis for long-lived humoral immunity.^10^ Bcl6 is essential for Tfh cell differentiation and germinal center (GC) development, and upregulation of CXCR5 promotes Tfh cell migration to the T-B boundary and localization to the GC.^10^^,^^11^ Tfh cells must be effectively induced by peptide vaccines to produce sustained high-affinity antibodies.^8^

T cell epitopes of carrier proteins with large molecular weights, such as keyhole limpet hemocyanin (KLH)^12^ and tetanus toxoid,^13^^,^^14^ have been used in vaccines. Due to their large molecular weight, antibodies against the carrier protein are produced instead, reducing vaccine effectiveness and causing an unexpected immune response.^3^ In addition, this may unexpectedly induce cellular immunity.^3^^,^^15^^,^^16^ To address these limitations, carrier peptides with a small number of amino acid bases must be screened.

Recently, Lu et al.^17^ identified SARS-CoV-2 spike (S) epitopes in patients with COVID-19. The S_864-882_ S epitope, which is related to circulating Tfh cells, was reported to be present in multiple human leukocyte antigen (HLA)s for T cell activation. This suggests that the S_864-882_ S epitope can be an efficient T cell epitope to induce Tfh cells, as a Tfh epitope, in peptide-based vaccines.

Angiotensin II (Ang II) is the main effector hormone of the renin-angiotensin-aldosterone system (RAS). RAS is known to be involved in multiple physiological features including regulation of blood pressure. Ang II, Asp-Arg-Val-Tyr-Ile-His-Pro-Phe, is cleaved from angiotensinogen, originated from liver, by renin and angiotensin-converting enzyme (ACE).^18^ Ang II increases blood pressure via activation of Ang II type 1 receptor (AT1R) signaling through a variety of physiological and pathophysiological mechanisms, including vasoconstriction and sodium and water retention.^19^ In the clinical field, antihypertensive drugs (i.e., ACE inhibitors or angiotensin receptor blockade) to target Ang II have become the major antihypertensive agents. Blocking Ang II binding to AT1R and inhibiting the RAS pathway may be the cornerstone of vaccine therapy for hypertension.^2^

In this study, we designed a peptide vaccine against angiotensin II (Ang II) by linking the Tfh epitope with angiotensin II (Tfh-AngII) and evaluated the efficiency of the Tfh-AngII vaccine in inducing Tfh cells and GC B cells and mitigating hypertension in a mouse model. Finally, we assessed whether the Tfh epitope activates human peripheral blood mononuclear cells (PBMCs) collected from healthy individuals with SARS-CoV-2 vaccine.

## Results

### SARS-CoV-2-derived follicular helper T cell epitope conjugated Ang II vaccine induced Ang II specific antibody production and Tfh cells in mice

Virus-specific T cells are important for effective immunity against SARS-CoV-2. Virus-specific T cells recognize peptides presented by MHC proteins on the surface of antigen-presenting cells (APC), and several potential CD4^+^ or CD8^+^ T cell epitopes have been identified in the S protein of SARS-CoV-2.^20^^,^^21^^,^^22^^,^^23^ Using the immune epitope database (IEDB) recommended 2.22 (http://tools.iedb.org/mhcii/), we found that the S_1007-1025_ sequence (YVTQQLIRAAEIRASANLA)^23^^,^^24^^,^^25^ from the SARS-CoV-2 spike protein showed a low percentile rank to H2-IAd (Table 1), MHC class II of BALB/c mice (percentile rank: 0.76; low percentile indicates high affinity). This suggests that S_1007-1025_ functions as a helper T cell (Th) epitope.

**Table 1:** Prediction of the binding of S1007-1025 for mouse MHC class II

Allele	Start	End	Sequence	Percentile rank
H2-IA^d^	1010	1023	QQLIRAAEIRASAN	0.76
	1008	1021	VTQQLIRAAEIRAS	1.3
	1009	1022	TQQLIRAAEIRASA	1.3
	1007	1020	YVTQQLIRAAEIRA	2
	1012	1025	LIRAAEIRASANLA	2.2
	1011	1024	QLIRAAEIRASANL	2.6
H2-IA^b^	1010	1023	QQLIRAAEIRASAN	3.2
	1009	1022	TQQLIRAAEIRASA	4.5
	1011	1024	QLIRAAEIRASANL	7.4
	1012	1025	LIRAAEIRASANLA	8.9
	1008	1021	VTQQLIRAAEIRAS	11
	1007	1020	YVTQQLIRAAEIRA	33

To date, there has been no reports of peptide-based vaccine using SARS-CoV-2 Spike protein-derived T epitope. Therefore, we examined whether the SARS-CoV-2 spike-derived T epitope functions as part of the Ang II vaccine. When the Th-Ang II vaccine was administered to mice three times, an anti-Ang II antibody was induced (Figure S1A), and Tfh and GC B cells significantly increased (Figure S1B, See also Figure S2). Increased numbers of Tfh cells are critical for a durable humoral immune response.^7^^,^^10^ This result can be regarded as proof that humoral immunity is activated, even when an endogenous B cell epitope is selected. Those data suggested that SARS-CoV-2 spike derived Th epitope functions as vaccine. Based on this result, we conducted the further experiments using Tfh epitope.

The Tfh cell epitope was previously identified in patients with COVID-19. S_864-882_ has been reported to activate Tfh cells in human PBMCs.^17^ We examined whether this Tfh epitope (S_864-882_ sequence, “LLTDEMIAQYTSALLAGTI”) could also function as a T cell epitope in mice. Using IEDB recommendation 2.22, we predicted that S_864-882_ could bind to MHC class II (Table 2). S_864-882_ showed a strong affinity for H2-IA. In particular, the 14 amino acid sequence (S_868-881_) consisting of “EMIAQYTSALLAGT” showed strong affinity (percentile rank: 9.1 for H2-IAd, and 8.4 for H2-IAb). The haplotype of BALB/cA mice contains H2-IAd, therefore, this sequence could potentially induce helper T cells in BALB/cA mice. The S_868-881_ from the SARS-CoV-2 spike protein might activate T cells in mice as a mouse T cell epitope.

**Table 2:** Prediction of the binding of S864-882 for mouse MHC class II

Allele	Start	End	Sequence	Percentile rank
H2-IA^d^	868	881	EMIAQYTSALLAGT	9.1
	867	880	DEMIAQYTSALLAG	14
	869	882	MIAQYTSALLAGTI	28
	866	879	TDEMIAQYTSALLA	36
	865	878	LTDEMIAQYTSALL	65
	864	877	LLTDEMIAQYTSAL	66
H2-IA^b^	869	882	MIAQYTSALLAGTI	7.3
	868	881	EMIAQYTSALLAGT	8.4
	867	880	DEMIAQYTSALLAG	16
	866	879	TDEMIAQYTSALLA	32
	865	878	LTDEMIAQYTSALL	68
	864	877	LLTDEMIAQYTSAL	73

Next, we evaluated whether S_868-881_, a Tfh epitope, functions as a T cell epitope in the vaccine to induce a humoral immune response in mice. We compared the immunogenic response of Tfh epitope-Ang II vaccine between C57BL/6 and BALB/c mice (Figure S3). Anti-Ang II antibodies were induced in both mice; however, as expected, the antibody titers were higher in BALB/c mice. Therefore, the BALB/c mice were selected for further experiments. Also, a group that received Ang II (no Tfh epitope) and adjuvant at the same time was also produced, but antibody titers did not increase in this group (Figure S4A). These antibody titers were dose-dependent (Figure S4B) when administered at different doses of Tfh- Ang II vaccine 100, 300, and 500 μg. In the previous report, the dose of vaccine was 1000 μg, which induced about 10^3^-10^4^ antibody titer and suppressed angiotensin II-induced hypertension.^26^ In this study, antibody titers comparable to that were obtained at 500 μg. Therefore, we determined that 500 μg was sufficient for the vaccine dose in this study, and administered the vaccine at this dose in subsequent experiments.

**Figure 1:**
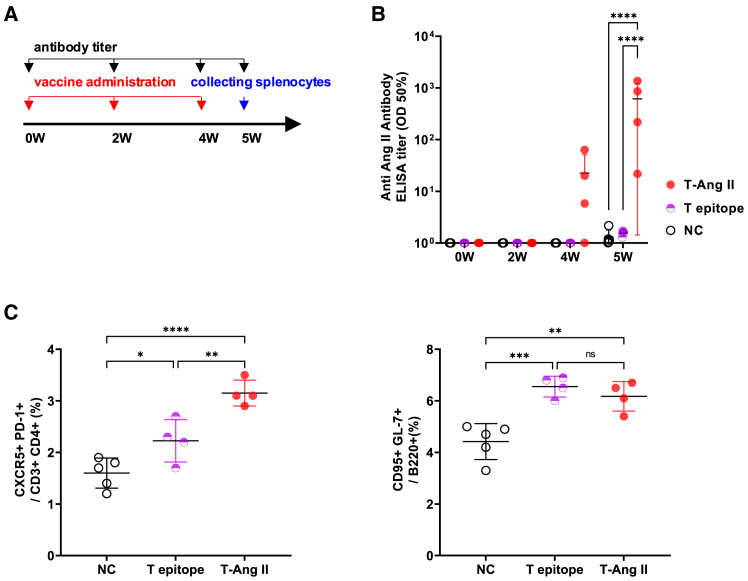
Follicular helper T (Tfh) epitope-angiotensin (Ang) II vaccine produced Ang II specific antibody, and induced Tfh cells, germinal center B cells (A) Experimental scheme to analyze vaccine-induced Tfh and GC B cells. BALB/c male mice were intradermally immunized with Tfh epitope or Tfh epitope -Ang II vaccine three times at 2 weeks interval. At 5 weeks after the first dose of vaccine, induction of Tfh and GC B cells in the spleen were evaluated using flow cytometry. Mice were divided into the following groups: Tfh-Ang II vaccine group (T-Ang II, *n* = 4), Tfh epitope only group (T epitope, *n* = 4), negative control group (NC, *n* = 5). (B) Anti-Ang II antibody titer measured by ELISA at 0, 2, 4, 5 weeks (W) after the first dose of vaccine. ∗∗∗∗*p* < 0.0001. (C) Percentage of Tfh cells and GC B cells in splenocytes, analyzed using flow cytometry. NC: negative control. T epitope: Tfh epitope, T-Ang II: Tfh-Ang II vaccine. ∗*p* < 0.05, ∗∗*p* < 0.01 GC, germinal center.

The Tfh-Ang II vaccine (T-Ang II) or Tfh epitope (T epitope) peptide was administered intradermally every 2 weeks, and anti-Ang II antibody titers were measured at 0, 2, 4, and 5 weeks after administration. At week 5, the spleens were collected and analyzed for Tfh and GC B cells using flow cytometry (Figure 1A). Anti-Ang II antibody titer increased at week 5 in the T-Ang II group (Figure 1B). Tfh and GC B cells were examined by flow cytometry (Figure 1C). Using flow cytometry, Tfh cells were defined as CXCR5+PD-1+ CD4^+^ T cells^7^^,^^27^^,^^28^ and GC B cells were defined as CD95+GL-7+ cells in B220 + B cells^29^^,^^30^ (See also Figure S2). The Tfh cell population was significantly higher in the T-Ang II group than in the negative control group (T-Ang II vs. NC, *p* < 0.0001). In addition, the Tfh cell population was significantly higher in the T epitope group than in the negative control group (T epitope vs. NC, *p* < 0.05). Similarly, the population of GC B cells was significantly increased in the T-Ang II group compared to that in the negative control group (T-Ang II vs. NC, *p* < 0.005). Even with the Tfh epitope alone, the number of GC B cells significantly increased compared to that in the negative control group (T epitope vs. NC, *p* < 0.001). These results suggest that the Tfh epitope successfully induced antibody production by effectively increasing the number of Tfh and GC B cells in mice.

### Tfh-Ang II vaccine produced anti-Ang II antibodies and provided an antihypertensive effect

**Figure 2:**
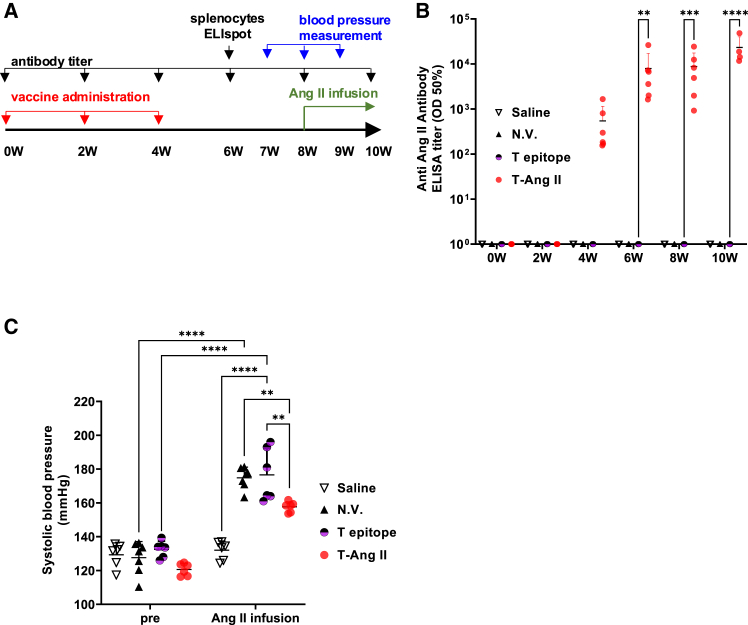
Tfh-AngII vaccine attenuated hypertension induced by Ang II infusion (A) Experimental scheme for evaluating therapeutic effect of Tfh epitope- Ang II vaccine on hypertension in an angiotensin II infusion mouse model. BALBc/A male mice were divided into the following groups: Tfh-Ang II vaccine group (T-Ang II, *n* = 6), Tfh epitope only group (T epitope, *n* = 6), Ang II loading without vaccine administration (No vaccine: N.V., *n* = 7), and a negative control group that received only saline (Saline, *n* = 6). Tfh epitope or Tfh epitope- Ang II vaccine was intradermally administered to mice with alum adjuvant thrice at interval of 2 weeks. At 8 weeks after the first dose injection, angiotensin II (0.7 μg/kg/min) was continuously infused with an osmotic mini pump for 2 weeks. Blood was collected every 2 weeks for antibody titer determination. Blood pressure was measured every week from the seventh week. (B) Ang II-specific IgG titer in the serum was analyzed using ELISA. ∗∗*p* < 0.005, ∗∗∗*p* < 0.001, ∗∗∗∗*p* < 0.0001, T epitope vs. T-Ang II. (C) Systolic blood pressure in response to Ang II infusion was measured in control, T epitope only, or Tfh-Ang II vaccine-immunized mice. The blood pressure at the start of continuous administration is noted as “pre”, and that after 1 week is noted as “Ang II infusion”. ∗∗*p* < 0.01, ∗∗∗∗*p* < 0.0001.

Next, we examined whether Tfh-Ang II vaccine-induced antibodies had an antihypertensive effect in an angiotensin II mouse model (Figure 2A). The Tfh epitope-Ang II vaccine (T-Ang II) or Tfh epitope (T epitope) group was administered three times every two weeks. In the T-Ang II group, antibody titers against Ang II increased after three doses of the vaccine and were significantly higher than those in the negative control and T epitope groups (Figure 2B). During the first week of continuous administration, systolic blood pressure increased to around 180 mmHg (174.9 ± 23.3 mmHg) in the unvaccinated group against 130 mmHg (132.1 ± 8.9 mmHg) in the saline group. In contrast, the vaccine-treated group had a low increase in blood pressure, which remained below 160 mmHg (157.6 ± 21.4 mmHg, pro T-Ang II vs. pro T epitope, *p* = 0.0037). There was no difference in systolic blood pressure when continuous Angiotensin II was started, but after administration, there was a significant reduction in systolic blood pressure in the T-Ang II group compared to the N.V. group (pro T-Ang II vs. pro N.V., *p* = 0.0073) (Figure 2C). Diastolic blood pressure also increased to 140 mmHg (137.2 ± 21.8 mmHg) in the unvaccinated group, while it remained around 120 mmHg (123.4 ± 16.0 mmHg) in the vaccine group (T-Ang II vs. N.V., *p* = 0.046). These results indicated that the Tfh epitope-Ang II vaccine promoted Ang II-specific antibody production and suppressed Ang II-induced hypertension in mice.

### T cell activation was induced by SARS-CoV-2-derived Tfh-epitope in immunized mice, but not angiotensin II

**Figure 3:**
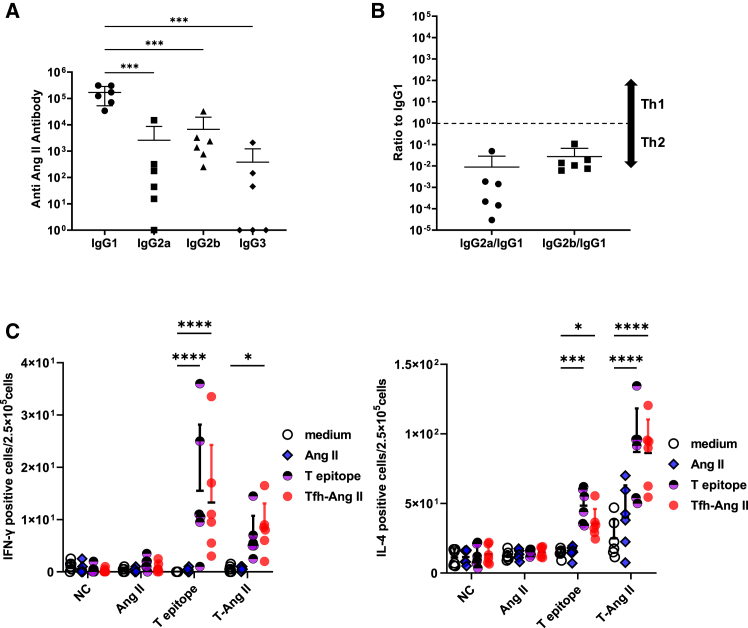
Ang II-specific cellular immune response was not induced by Tfh-Ang II vaccine (A and B) IgG subclass (IgG1, IgG2a, IgG2b, and IgG3) for Ang II titer and ratio to IgG1(IgG2a/IgG1, IgG2b/IgG1) analyzed using ELISA. Serum extracted at 8 weeks after the first dose of vaccine was used for analysis. ∗∗∗*p* < 0.001, vs. IgG1. (C) Tfh epitope vaccine response to splenocytes from immunized mice (ELISpot). Tfh epitope- Ang II (T-Ang II, *n* = 6), Tfh epitope only (T epitope, *n* = 6), Ang II only (Ang II, *n* = 6) and Negative Control (NC, *n* = 6). Each peptide was administered three times every two weeks. 5 weeks after the first dose, splenocytes were collected and ELISpot was performed. Medium, Ang II, Tfh epitope (T epitope), and Tfh epitope- Ang II vaccine (Tfh- Ang II) peptides were used for stimulation in ELISpot. IFN-γ positive spots, ∗*p* < 0.05, ∗∗∗∗*p* < 0.0001, vs. medium. IL-4 positive spots, ∗*p* < 0.05, ∗∗∗*p* < 0.001, ∗∗∗∗*p* < 0.0001, vs. medium.

We investigated T cell activation in Tfh epitope-Ang II vaccine immunised mice using IgG subclass ELISA and enzyme-linked immunosorbent spot (ELISpot) assay. The IgG subclass was examined by ELISA using sera from mice immunized with the Tfh epitope Ang II vaccine at 8 weeks. The production of antibodies was biased toward IgG1 (Figure 3A). The ratios of the antibody titer subclass IgG2a and IgG2b to IgG1 were also less than 1.0, so they were biased toward IgG1 (Figure 3B). This result indicates that the antibodies produced by the Tfh epitope vaccine were biased toward the Th2.

To evaluate the risk of activation of auto-reactive T cells induced by Tfh epitope-Ang II vaccine, we examined cytokine production (IFN-γ and IL-4) of splenocyte from immunized mice using ELISpot assay. As the results, the vaccine-administered group produced higher levels of IFN-γ and IL-4 than the wild type (Figure 3C, See also Figure S5). These data suggest that the vaccine conjugated with the Tfh epitope did not activate autoreactive T cells specific to Ang II.

### The vaccine efficacy of Tfh- Ang II was enhanced by priming with SARS-CoV-2 spike protein

**Figure 4:**
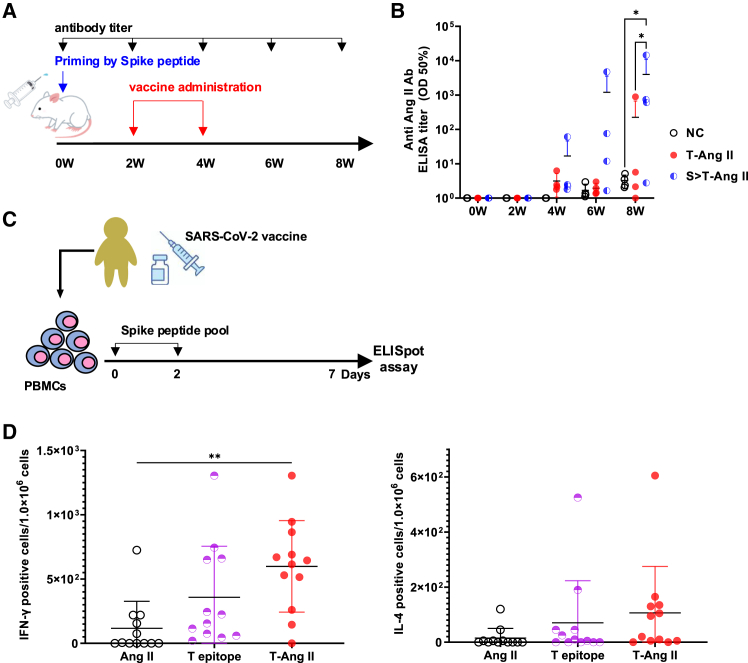
Spike priming enhanced Tfh-AngII vaccine-induced immune responses in mice and human PBMCs (A) Experimental scheme to analyze the priming effect by SARS-CoV-2 Spike protein. BALB/c male mice were intradermally immunized with Tfh-Ang II vaccine twice at 2 weeks interval. The priming group(S > T-Ang II, *n* = 4) received injection 0.5 μg of SARS-CoV-2 recombinant protein before 2 weeks from the vaccine. No priming and Tfh-Ang II vaccine administration group (T-Ang II, *n* = 4), Negative control group (NC, *n* = 4). (B) Anti-Ang II antibody titer measured by ELISA at 2 weeks interval. ∗*p* < 0.05. (C) Experimental scheme for evaluating activated PBMCs using ELISpot assay. PBMCs collected from SARS-CoV-2 mRNA vaccines (Pfizer/BioNTech) injection at 6 weeks. PBMCs were stimulated with 10 μg/mL SARS-CoV-2 spike peptide pool twice at days 0 and 2. On day 7, the PBMCs were stimulated with medium, Ang II (10 μg/mL), Tfh epitope (10 μg/mL), and Tfh- Ang II (10 μg/mL). IFN-γ and IL-4 secretion by the PBMCs were analyzed using ELISpot assay. (D) IFN-γ and IL-4 secreting PBMCs were counted in each well. The counted spots in stimulated groups were subtracted from background (medium). ∗∗*p* < 0.01, vs. Tfh-AngII. Ang, angiotensin; Tfh, follicular helper T.

We expected that the Tfh epitope-specific T cells would be activated upon priming with the SARS-CoV-2 spike protein during COVID-19 vaccination or infection, leading to the enhancement of Tfh epitope-Ang II vaccine efficacy. We evaluated whether the anti-Ang II antibody titer was enhanced by priming with the SARS-CoV-2 spike protein (Figure 4A). Balb c/A mice were primed with SARS-CoV-2 S recombinant protein, administered Mouse Tfh epitope-Ang II vaccine 2 and 4 weeks later, and we evaluated antibody titers against Ang II every 2 weeks. This Anti Ang II antibody titer of the primed with S-protein group (S > T-Ang II) was compared with that of the group that was not primed (T-Ang II). S > T-Ang II group had higher antibody titers than T-Ang II group and this tendency was stronger over time (Figure 4B, ∗*p* < 0.05). At week 8, the antibody titers in S > T-Ang II group were around 4×10^3^ (OD50%, 3,960 ± 6,085) compared to around 2×10^2^ (OD50%, 224 ± 383) in the T-Ang II group.

To also examine in humans whether priming with SARS-CoV-2 spike protein enhanced T cell response, we used human PBMCs collecting from 12 randomized human donors. The donors were twice administered vaccines against COVID-19, then collected PBMCs after 6 weeks. We stimulated the PBMCs with SARS-CoV-2 spike protein peptide pool and then analyzed with ELISpot assay (Figure 4C). The number of the IFN-γ and IL-4 producing T cells in response to Ang II, which is the B cell epitope of our vaccine, were not increased compared to the negative control. In contrast, the number of IFN-γ producing T cells were increased by Tfh-epitope stimulation in the stimulation groups (Figure 4D, ∗∗*p* < 0.005). IL-4 producing T cells also increased, although the difference was not statistically significant.

## Discussion

In this study, we designed a peptide-based vaccine by conjugating the SARS-CoV-2 S-derived Tfh-epitope S_864-882_, with angiotensin II as the B-cell epitope to form the Tfh epitope-Ang II peptide vaccine. The Tfh epitope- Ang II vaccine successfully induced specific anti Ang II antibody production and increased the number of Tfh and GC B cells in the spleen (Figures 1 and 2). The Tfh epitope was originally identified from SARS-CoV-2 S protein as a universal epitope that activates Tfh cells in multiple donors.^17^ Tfh cells are an essential subset of T cells for antibody production and the interaction between Tfh cells and GCB cells is key in the production of high affinity antibodies.^31^^,^^32^ The increase in these cells with administration of the vaccine conjugating the Tfh epitope and endogenous protein suggests that the Tfh epitope has a directivity to activate Tfh cells, which generated antibodies against endogenous protein with high affinity. The antibodies against the endogenous protein (in this case, Ang II) were effective (Figure 2C), indicating the future potential of vaccine therapy against endogenous proteins.

The IgG subclass of anti-Ang II antibodies was heavily biased toward IgG1 by the Tfh epitope-AngII vaccine (Figures 3A and 3B). Within GCs, Tfh cells have been reported to promote B-cell activation and differentiation, mainly through the secretion of IL-21 and co-stimulation of CD40L. IL-21 induces class switching to IgG1 and IgG3 by naive B cells and increases the secretion of these Ig isotypes by memory B cells.^33^^,^^34^ The Tfh epitope probably activated Tfh cells and caused this induction of directionally to IgG1. Additional investigations are required to evaluate the extent to which the Tfh epitope-Ang II vaccine affects the Th1/Th2 balance.

We found that the splenocytes of mice administered Tfh epitope or Tfh epitope-Ang II vaccine, produce IFN-γ responsive to these epitopes (Figure 3C). In the experiment using human PBMCs, cells producing IFN-γ in response to Tfh epitope were also observed (Figure 4D). Recently, Tfh cells have been reported to have multiple subsets^35^^,^^36^ and Tfh cells in the GC can also express other cytokines characteristic of Th cells, such as IFN-γ^11^^,^^37^ and IL-4.^37^^,^^38^ IFN-γ producing Tfh cells in GCs are a subset of Tfh cells with a history of T-bet expression and are thought to produce IFN-γ in a STAT4 signaling-dependent manner.^39^^,^^40^ They are known to promote humoral immune signaling in type 1 responses.^41^ The production of IFN-γ by Tfh epitope stimulation in this study may suggest that antibody production is enhanced by the activation of these signals. It should be added, however, that these biases are also influenced by adjuvants. In this study, phosphate aluminum was mixed as an adjuvant and administered along with the Tfh epitope-Ang II vaccine to enhance the humoral immune response. Tfh cells can be induced using an alum adjuvant.^42^ It is possible that the Tfh epitope and adjuvant synergistically activate Tfh cells, leading to beneficial results.

SARS-CoV-2 was spread worldwide from China, causing the COVID-19 pandemic since December 2019.^43^ As of May 9, 2024, the World Health Organization reported 775,364,261 confirmed cases of COVID-19 and that 13.59 billion doses of COVID-19 vaccines were administered (https://covid19.who.int). It was reported that 100% of CD4^+^ T cells were re-stimulated with S pools in an analysis with PBMCs from convalescent patients with COVID-19. Approximately 70% of CD4^+^ T cells were detected in PBMCs from unexposed individuals.^44^ Moreover, most vaccines developed using various platforms, such as inactivated viruses, viral vectors, and nucleic acids, target the SARS-CoV-2 spike protein.^45^ Individuals in this study were primed by the SARS-CoV-2 spike protein, making the vaccine in this study effective. Indeed, our results showed that the antibody titers from this vaccine were significantly elevated when primed with a recombinant viral spike protein in mice (Figures 4A and 4B). Previous reports have shown increases in antibody titers due to such priming proteins. For example, multifunctional CD4^+^ and CD8^+^ T cells against *Mycobacterium tuberculosis* are induced when primed with specific recombinant *Bacillus* Calmette-Guérin and boosted with plasmid DNA as a strategy to prevent tuberculosis.^46^ Besides, DNA vaccines against the herpes simplex virus elicit stronger immune responses when combined with recombinant proteins derived from baculoviruses that express D-1.^47^ Antibody titers against SARS-CoV-2 tend to be higher than normal after vaccination if the patient has previously been infected with COVID-19.^48^^,^^49^^,^^50^ These reports indicate that in vaccines targeting viruses, administration in combination with recombinant proteins at specific sites can induce strong immune responses. It is also believed that robust Tfh and GCB cell activation is behind such prime-boost immunization, and the Tfh epitope activating Tfh cells we developed in this study is more likely to induce such a response.

In addition, human PBMCs collected from volunteers immunized with the mRNA vaccine were activated by the Tfh epitope-Ang II vaccine (Figures 4C and 4D). The human PBMCs used in this experiment were originally collected at random, that is, they each have their own HLA. In spite of this, it is very interesting that this Tfh epitope activated human PBMCs and caused them to release cytokines. These results led us to presume that the Tfh epitope-based vaccine might be more effective in several populations who had COVID-19 vaccine or infection. The Tfh epitope used in this study has been described as activating multiple Tfh clonotypes,^17^ and our experimental results support this. Notably, although the B cell epitope is an endogenous protein, angiotensin II in this study, Tfh epitope-Ang II vaccine induced more robust production of Ang II specific IgG, which has neutralizing activity. Therefore, Tfh cells and the T cell epitope that activates them are particularly important for priming with vaccines, which is a very important result for future vaccine strategies. However, a detailed evaluation using flow cytometry and other methods is needed to determine whether Tfh cells are induced by the Tfh epitope-Ang II vaccine in human PBMCs.

In conclusion, SARS-CoV-2-derived T epitope functioned as a T cell epitope of peptide-based vaccine in the present study and could be useful for future vaccine development. The results of experiments with Tfh epitopes suggested that the epitope peptides could function as T cell epitopes in a vaccine by conjugating with B cell epitopes, such as Ang II peptides in mice.

### Limitations of the study

In this study, we utilized Tfh epitope derived from human samples.^17^ We confirmed that Tfh epitope conjugated with angiotensin II responded to produce antibody production, leading to anti-hypertensive effect in mouse model. However, how the Tfh-AngII in this study reacts in human MHC class II specifically or generally needs to be further investigated in human MHC class II transgenic mice or human clinical studies.

## Resource availability

### Lead contact

Further information and requests for resources and reagent should be directed to and will be fulfilled by the lead contact, Hiroki Hayashi (hayashih@cgt.med.osaka-u.ac.jp).

### Materials availability

This study did not generate new unique reagents.

### Data and code availability

• This study did not generate any large dataset.
• Any additional information in this study will be available from the lead contact upon reasonable request.

## Acknowledgments

This study was supported by 10.13039/501100001691JSPS 10.13039/501100001691KAKENHI (grant number 21H02827). We thank all the members of the Department of Health Development and Medicine for supporting this project. We thank Ms. Satoe Kitabata for secretary support and Jiao Sun and Yuka Yanagida for technical support.

## Author contributions

S.B. and H.N. designed and conducted the research; S.B., H.H., S.Y., and M.S. conducted the experiments and acquired the data; S.B., H.H., S.Y., N.Y., M.S., R.M., H.R., H.N., and K.Y. analyzed the data; and S.B., H.H., M.S., and H.N. wrote and edited the manuscript.

## Declaration of interests

The Department of Health Development and Medicine is an endowed department supported by the AnGes, 10.13039/100021023Daicel, and FunPep. The Department of Gene and Stem Cell Regenerative Therapy is an endowed department supported by AS Medical Support. The Department of Clinical Gene Therapy is an endowed department supported by 10.13039/100004336Novartis, AnGes, 10.13039/501100005612Shionogi, Boeringher, Fancl, Saisei Mirai Clinics, 10.13039/100031953ROHTO, and FunPep. R.M. is a stockholder and scientific adviser for FunPep and AnGes. H.N. is a stockholder and scientific adviser for FunPep. The funders provided salaries to the authors. However, they did not play any additional roles in the study design, data acquisition and analysis, or manuscript preparation.

## STAR★Methods

### Key resources table

**Table:** 

REAGENT or RESOURCE	SOURCE	IDENTIFIER
**Antibodies**		

APC Cy7 anti-mouse CD3	BD Biosciences	Cat# 560590; RRID:AB_1727461
FITC anti-mouse CD4	BD Biosciences	Cat# 561828; RRID:AB_395013
PerCP Cy5.5 anti-mouse CD279	BioLegend	Cat# 135208; RRID:AB_2159184
PE Cy7 anti-mouse CD185	BioLegend	Cat# 145516; RRID:AB_2562209
PerCP Cy5.5 anti-mouse CD45R/B220	BioLegend	Cat# 103236; RRID:AB_893354
PE anti-mouse CD95	BioLegend	Cat# 152608; RRID:AB_2632902
FITC anti-mouse GL7 antigen	BioLegend	Cat# 144604; RRID:AB_2561696
Anti-Mouse IgG (HRP)	Cytiva	Cat# NA931-1ML
Anti-Mouse IgG1 (HRP)	Abcam	Cat# ab97240
Anti-Mouse IgG2a heavy chain(HRP)	Abcam	Cat# ab97245
Anti-Mouse IgG2b heavy chain(HRP)	Abcam	Cat# ab97250
Anti-Mouse IgG2c heavy chain(HRP)	Abcam	Cat# ab97255
Anti-Mouse IgG3 heavy chain(HRP)	Abcam	Cat# ab97260

**Biological samples**		

Human volunteers	Osaka university	N/A

**Chemicals, peptides, and recombinant proteins**		

Zombie Violet™ Fixable Viability Kit	BioLegend	Cat# 423114
mouse Anti-Mouse CD16/32 (Mouse Fc Block)	BD Biosciences	Cat# 553142
SARS-CoV-2 (Spike Glycoprotein SUB1)	PepMix	Cat# PM-WCPV-SU1-1
SARS-CoV-2 (Spike Glycoprotein SUB2)	PepMix	Cat# PM-WCPV-SU2-1
Recombinant Human IL-2	PeproTech	Cat# 200-02-10UG
RPMI-1640 with L-glutamine and phenol red	Gibco	Cat# 11875-093
Fetal Bovine Serum (FBS)	SIGMA	Cat# 173012
Penisillin/streptomycin	nakalai tesque	Cat# 26253-84
ACK erythrocyte-lysing buffer	Gibco	Cat# A1049201
Stain Buffer (FBS)	BD Biosciences	Cat# 554656

**Software and algorithms**		

Prism version 9.5.1	Graphpad	https://www.graphpad.com/features

### Experimental model and study participant details

#### Animals

Male BALB/c and C57BL/6 mice (6–8-week-old) were purchased from CLEA Japan, Inc. (Tokyo, Japan). All mice were maintained under light/dark cycles every 12 h.

#### Ang II infusion model

The mice were divided into Tfh epitope- Ang II vaccine group (T-Ang II, 500 μg, *N* = 6), positive control group that received only the carrier protein Tfh epitope (T epitope, 500 μg, *N* = 6), the group that received no vaccine, but continuous administration of Ang II (N.V., *N* = 7), and negative control group (Saline, *N* = 6), which did not experience hypertension and received a continuous injection of saline solution. The vaccine and peptide were administered three times every 2 weeks to male BALBc/A mice (6–8-weeks-old), and the titer of anti-Ang II antibodies was measured every 2 weeks. Ang II (0.7 μg/min/kg) was administered continuously for 1 weeks starting at week 8 (4 weeks after the last vaccine administration) through an osmotic mini-pump (Muromachi Kikai Corporation, Tokyo, Japan).

Blood pressure was measured at 7, 8, and 9 weeks using the tail-cuff method (BP-98A, SOFTRON, Tokyo, Japan). Systolic blood pressure was measured thrice for each individual. A graph plotting the mean of the blood pressure measured 3 times at each time point (pre = 8W, the start of continuous administration, and Ang II infusion = 9W, 1W later) was drawn and analysed statistically (2-way ANOVA).

#### Human subjects for PBMCs collection

The vaccine study protocol was approved by the Osaka University Institutional Review Board (IRB) (reference no. 21487). Informed consent was obtained from all the participants before sample collection. PBMCs were collected three weeks after the administration of two doses of the Pfizer mRNA vaccine (Corminaty).

### Method details

#### Peptides and immunization

Tfh epitope-Ang II and Tfh epitopes were synthesised by the Peptide Institute, Inc. (Osaka, Japan). These peptides with an aluminium adjuvant (InVivoGen) were intradermally administered to BALB/c mice three thrice at an interval of 2 weeks. Serum samples were collected every 2 weeks for antibody titer determination or other analyses.

#### Antibody titer determination via ELISA

Anti-Ang II antibody titers were measured by the following method, referring to previous reports.^26^ Bovine serum albumin (Peptide Institute)-conjugated Ang Ⅱ dissolved in carbonate buffer (10 μg/mL) was coated on a 96-well plate on the first day. On the second day, the wells were blocked with the blocking buffer (PBS Tween-20 0.05% (PBS-T) containing 5% skim milk) for 2 h at room temperature (15°C–25°C). The sera were diluted 100-fold to 312,500-fold in the blocking buffer, added to the plates, and incubated overnight at 4°C. The following day, the wells were washed with PBS-T and incubated with HRP-conjugated anti-mouse IgG antibody (GE Healthcare) for 3 h at room temperature. For IgG subclass determination, HRP-conjugated anti-subclass IgG antibodies (IgG1, IgG2a, IgG2b, and IgG3; Abcam) were used as secondary antibody. These secondary antibodies were diluted 1000-fold in blocking buffer. After washing with PBS-T, the wells were incubated with the peroxidase chromogenic substrate, 3,3ʹ−5,5ʹ-tetramethyl benzidine (Sigma-Aldrich), for 30 min at room temperature, and the reaction was stopped using dilute sulfuric acid. Half maximum titer (Optical density (OD) 50% (450 nm)) was measured on a microplate reader (Bio-Rad Inc., Hercules, California, USA). The antibody titer is expressed as the serum dilution that exhibited half-maximal binding. The half-maximal binding was calculated as follows: absorbance was measured in each ELISA at concentrations from 100-fold to 312,500-fold and converted to logarithmic values of Half maximum titer. A nonlinear regression graph (sigmoid curve) of the Half maximum titer was created to determine the concentration at the 50% of maximum absorbance point and converted from logarithmic to real. When plotting into the graph, we entered 10° for those antibody values that were 0 because the Y-axis is in power of 10 notations.

#### ELISpot assay

ELISpot assay was performed based on the previous report.^26^^,^^51^ Mouse splenocytes were used for the assay following the manufacturer’s instructions (R&D Systems). The spleen tissues were mashed using two cell strainers (70 and 40 μm) to make a single cell solution of splenocytes, and the solution was centrifuged at 400 g for 5 min at 4°C to remove the supernatant. Erythrocytes were removed by lysing with 5 mL of ACK erythrocyte-lysing buffer (Gibco, Grand Island, NY, USA) for 3 min at room temperature; then, RPMI 1640 containing 10% FBS, 0.1% 2-mercaptoethanol, and 1% penicillin/streptomycin was added. Filtration plates (96-well plates, MERCK) were incubated with IFN-γ and IL-4 capture antibodies at 4°C overnight. The wells were washed with PBS-T and blocked with a blocking buffer (1% BSA, 5% Sucrose in PBS) for 2 h at room temperature. Splenocytes were isolated from the vaccinated spleen and 2 × 10^5^ cells with culture media (RPMI-1640) were added to the plate. Each group was also stimulated for 48 h with the following peptides: Tfh-Ang II and Tfh epitope peptides at 10 μg/mL each, with RPMI-1640 medium as the negative control and PMA and ionomycin both mixed 200 ng/mL as the positive controls. The wells were washed with PBS-T, incubated overnight with a detection antibody against mouse IL-4 or IFN-γ, and then incubated in buffer containing streptavidin-AP for 2 h. After washing, the cells were treated with 5-bromo-4-chloro-3-indolylphosphate/nitro blue tetrazolium solution (BCIP/NBT) for 30 min at room temperature, rinsed with deionised water, dried at room temperature, and the number of spots appearing was counted using fluorescence microscopy.

For ELISpot assay using human PBMCs, frozen PBMCs were thawed and washed with RPMI-1640. These PBMCs were collected from blood samples of 12 human participants 6 weeks after mRNA vaccination against COVID-19. The PBMCs were stimulated with SARS-CoV-2 peptide pool SUB1 and SUB2 (PepMix) at 1 μg/mL each. The stimulation solution contained human IL-2 (1 ng/mL; PeproTech). Stimulation was performed for 1 week, with the stimulation solution added on days 0 and 2. PBMCs were collected and divided into groups. An ELISpot assay kit (Cellular Technology Limited, USA) was used to detect IL-4 and IFN-γ in activated PBMCs according to the manufacturer’s instructions. Activated polyvinylidene-membrane 96-well plate was coated with anti-IL-4/IFN-γ capture antibodies. The next day, the membranes were washed with PBS. PBMCs (1 × 10^6^) were added to wells and re-stimulated with Ang II, Tfh epitope, and Tfh-Ang II peptides at 10 μg/mL each, with medium RPMI-1640 medium as the negative control and PMA and ionomycin 200 ng/mL as the positive control at 37°C for 48 h. After washing with PBS, the wells were incubated with anti-human IL-4/IFN-γ detection antibodies at room temperature for 2 h. IL-4/IFNγ spots were measured using an ImmunoSpot S6 analyser (Cellular Technology Limited). In creating the graph, the value for each sample was defined as the count value minus the negative control (culture medium) value. Samples with negative values were defined as 0 and plotted.

#### Flow cytometry for detection of Tfh, GC B cells, and cytokine secreted by CD4^+^ T cells

Analysis based on flow cytometry of Tfh and GCB cells and gating methods were performed in reference to previous reports.^28^^,^^29^ Male BALB c/A mice (6–8-week-old) were intradermally administered the vaccine or T epitope peptide thrice every 2 weeks. One week after the last administration of the vaccine, spleens were collected, and splenocytes were used to detect Tfh cells and GC B cells using flow cytometry.

The spleen tissues were mashed using two cell strainers (70 and 40 μm) to make a single cell solution of splenocytes, and the solution was centrifuged at 400 g for 5 min at 4°C to remove the supernatant. Erythrocytes were removed by lysing with 5 mL of ACK erythrocyte-lysing buffer (Gibco, Grand Island, NY, USA) for 3 min at room temperature; then, RPMI 1640 containing 10% FBS, 0.1% 2-mercaptoethanol, and 1% penicillin/streptomycin was added. The samples were centrifuged at 400 × g for 5 min at 4°C, supernatant was removed, and splenocytes were suspended in RPMI and counted. Approximately 2 × 10^6^ cells were used for staining. The cells were separated into FACS Buffer (BD Bioscience) for Flow cytometry. The Fc receptors were blocked by incubation with anti-mouse CD16/32 (mouse Fc receptor blocker; BD Biosciences) on ice for 30 min. Dead cells were stained with ZOMBIE Violet (Zombie Violet™ Fixable Viability Kit; BioLegend). A solution of fluorescently labelled antibodies was prepared and cells were stained at 4°C for 30 min. The following antibodies were used for staining: APC Cy7 anti-mouse CD3 (17A2) (BD Biosciences), FITC anti-mouse CD4 (GK1.5) (BD Biosciences), PerCP Cy5.5 anti-mouse CD279 (29F.1A12) (BioLegend), and PE Cy7 anti-mouse CD185 (L138D7) (BioLegend) for the analysis of Tfh cells. PerCP Cy5.5 anti-mouse CD45R/B220 (RA3-6B2) (BioLegend), PE anti-mouse CD95 (SA367H8) (BioLegend), and FITC anti-mouse GL7 antigen (GL7) (BioLegend) were used for GC B cell analysis. Flow cytometric analysis was performed using a BD FACS Aria III (BD Biosciences) and the results were analysed using a BD FACS DiVA (BD Biosciences).

#### PBMC collection from healthy vaccinees

The PBMCs were separated from whole blood using BD vacutainer ® CPT™ cell separation tube (BD). Briefly, whole blood was centrifuged at 1,600 × g for 15 min. The layer containing the PBMCs was collected and washed with PBS. The PBMCs were counted and stored in liquid nitrogen until further use.

#### Animal study approval

All animal experiments were approved by the Ethical Committee for Animal Experiments of the Osaka University Graduate School of Medicine.

### Quantification and statistical analysis

#### Statistical analysis

All graphs are expressed as means ± standard error of the mean. Differences in multiple samples were assessed via post hoc analysis using Tukey’s multiple comparison test. All statistical analyses were performed using Prism 8 software (GraphPad Software). *p* values were considered statistically significant at *p* < 0.05.
