# Supplementary material for: Development of a peptide-based vaccine using a T cell epitope derived from SARS-CoV-2
Source: iScience. 2025 Apr 28;28(6):112542. doi: 10.1016/j.isci.2025.112542 (PMC12141072; doi:10.1016/j.isci.2025.112542)
Supplement: Document S1. Figures S1–S5 [file mmc1.pdf]

iScience, Volume *28*

Supplemental information

Development of a peptide-based vaccine

using a T cell epitope derived from SARS-CoV-2

Satoshi Baba, Hiroki Hayashi, Shota Yoshida, Nanxiang Yin, Munehisa Shimamura, Ryuichi Morishita, Hiromi Rakugi, Hironori Nakagami, and Koichi Yamamoto

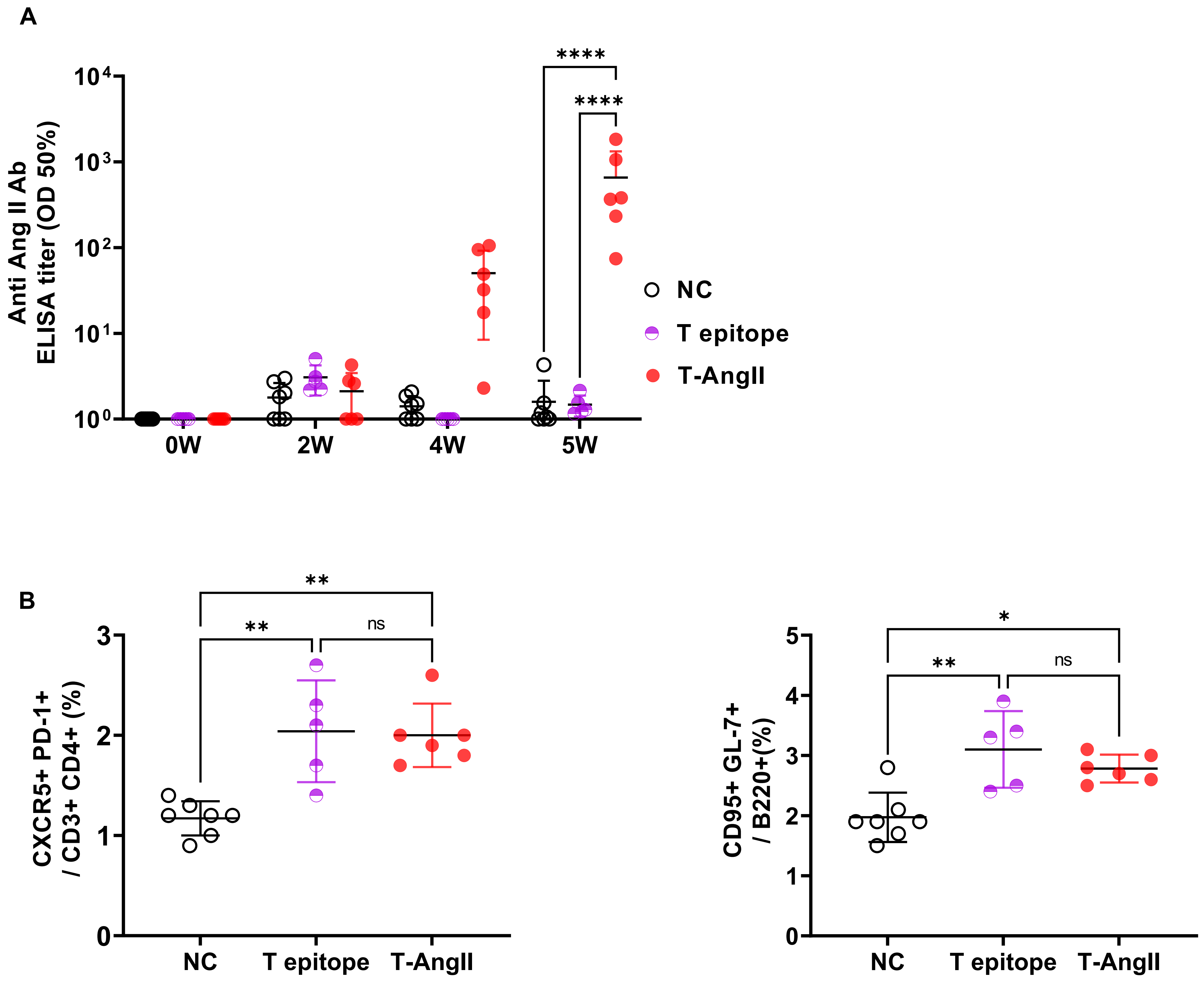

Figure S1. Angiotensin II vaccine using mouse T epitope derived from SARS-CoV-2 spike protein induced antibody production, and increased Tfh/GC B cells. Related to Figure 1.

(A) Anti-Ang II antibody titer measured by ELISA. NC: negative control, T epitope: Mouse Th epitope, T-Ang II: Th-Ang II vaccine. ****p<0.0001

(B) Tfh cells, GCB cells increase analyzed by flow cytometry after administration of T cell epitope for mice or the epitope - Ang II vaccine. *p<0.05, **p<0.01

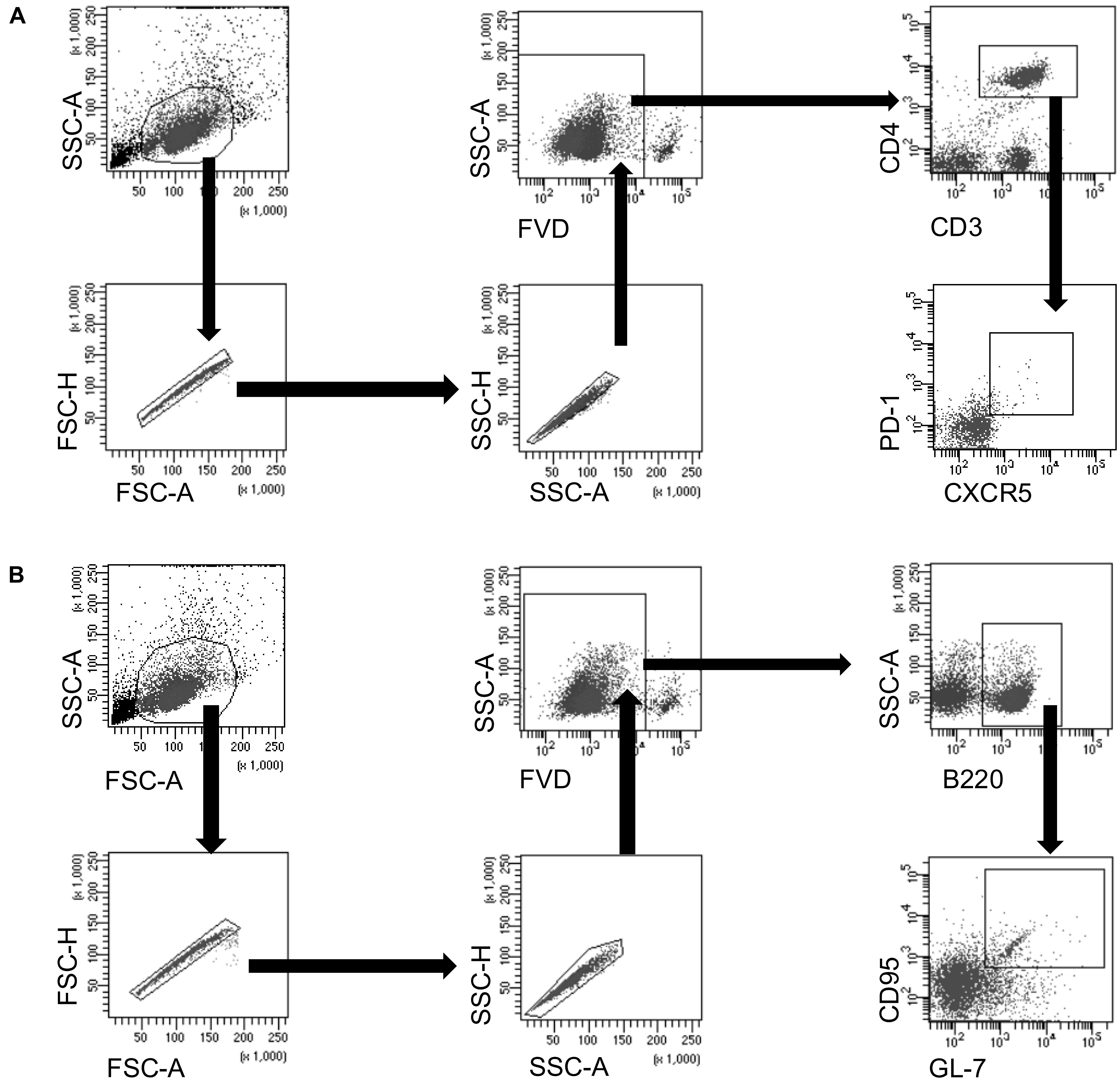

Figure S2. Gating strategies for flow cytometry analysis. Related Figure 1C and Figure S1B.

(A) Gating strategy for analysis of Tfh cells in flow cytometry. Tfh cells were stained and evaluated by flow cytometry. After gating CD3+CD4+ as helper T cells from singlet live lymphocytes, Tfh cells were gated with PD-1+CXCR5+ cells.

(B) Gating strategy for analysis of GC B cells in flow cytometry. After gating B220+ cells as B cells from singlet live lymphocytes, GC B cells were gated with CD95+GL-7+ cells.

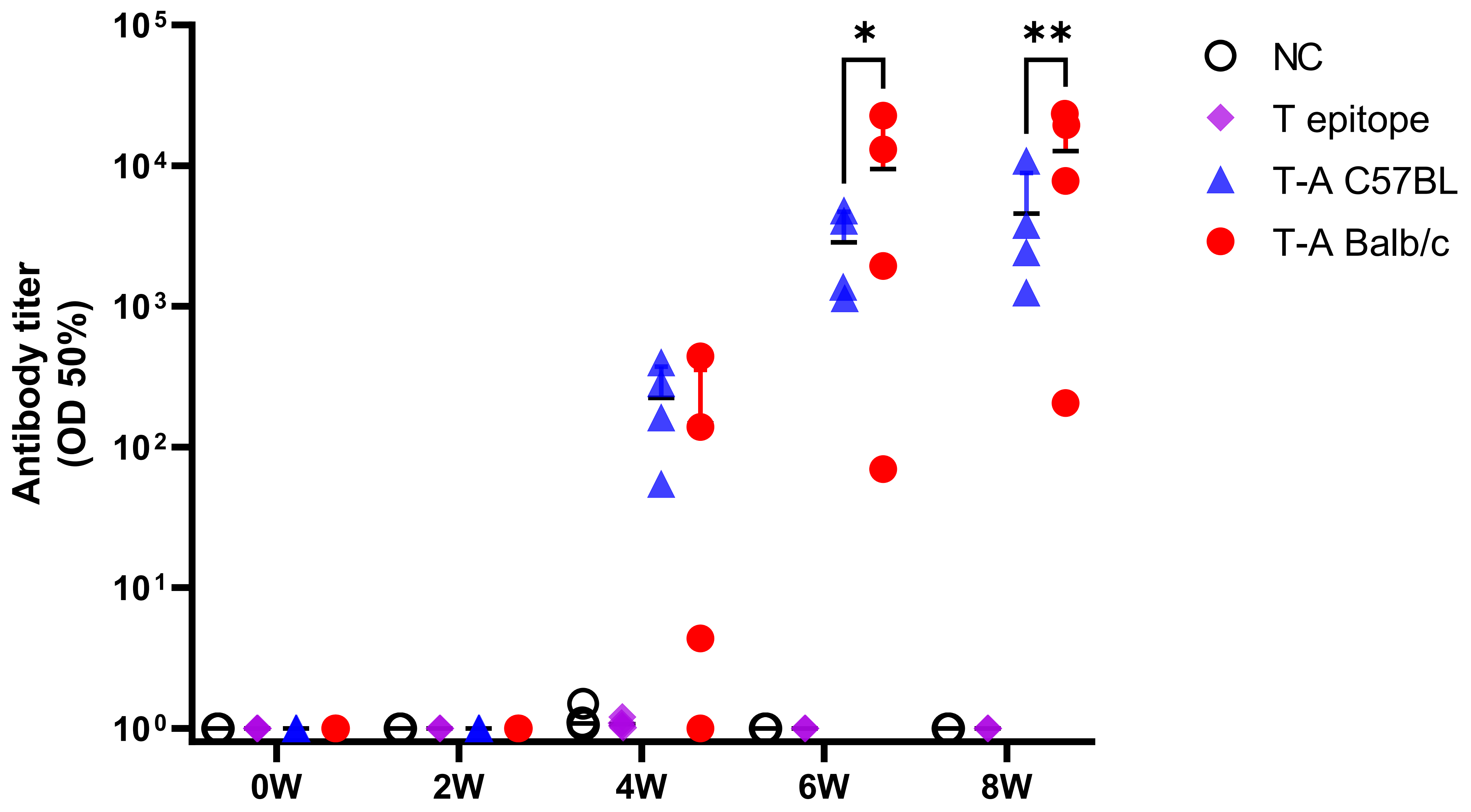

Figure S3. Comparison of immunogenic response of AngII vaccine conjugated with S864-882 (Tfh-AngII) between C57BL mice and Balb/c mice. Related to Figures 1-3.

C57BL(n=4) and Balb/c mice(n=4) were administered with each of Tfh-AngII (T-A) vaccine 500 µg three times every 2 weeks. Negative control (NC) : No vaccination (n=4). T epitope: Tfh epitope-only peptide without AngII (n=4). Anti-AngII antibody titers were measured by ELISA every 2 weeks through 8 weeks. *p<0.05, **p<0.01, Balb/c vs C57BL

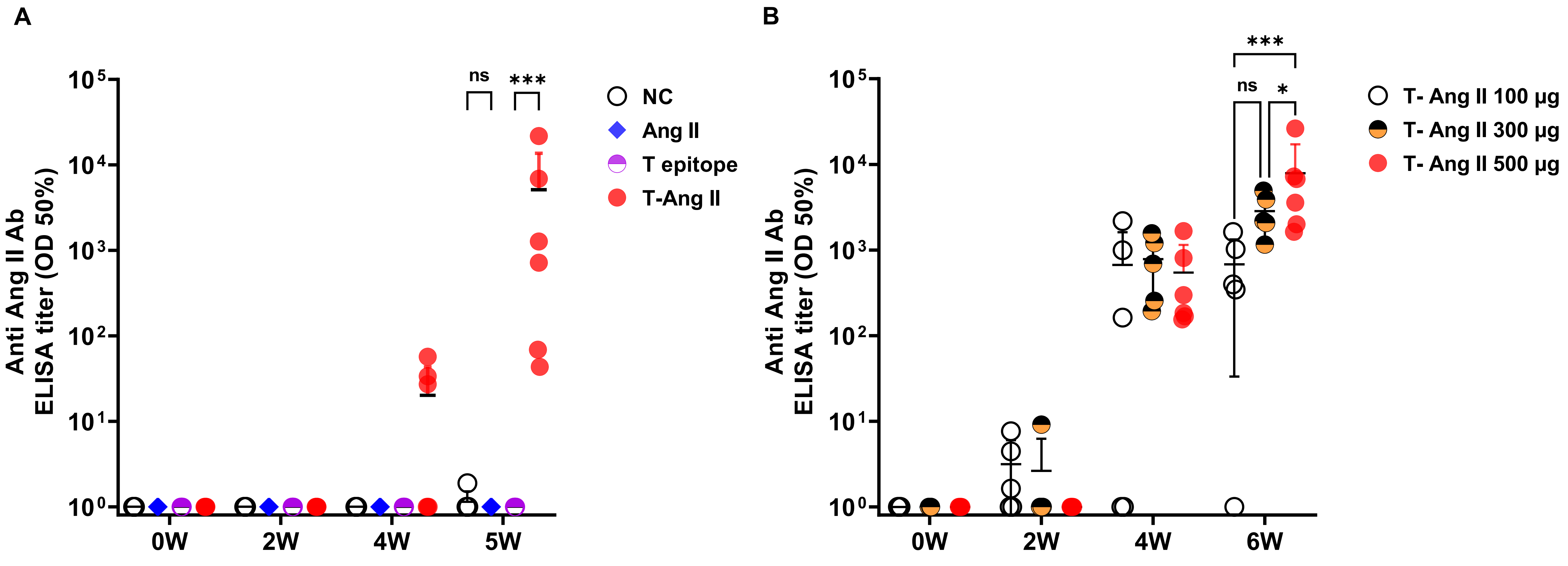

Figure S4. Confirmation of angiotensin II antigenicity and dose dependency of Tfh-AngII vaccine. Related to Figure 1.

(A) Administration of angiotensin II and alum alone does not produce anti-Ang II antibodies.

Balb/c mice, N=6 each, were administered peptides three times every 2 weeks; Tfh-AngII (T- Ang II) vaccine, Tfh epitope(T epitope), angiotensin II + alum (Ang II). Anti-AngII antibody titers were measured by ELISA every 2 weeks through 5 weeks.

(B) Dose dependency of the Tfh – Ang II vaccine (T- Ang II).

Balb/c mice ,N = 5-6 each, were administered three doses of T-Ang II in different concentrations (100μg, 300μg, 500μg) every two weeks.

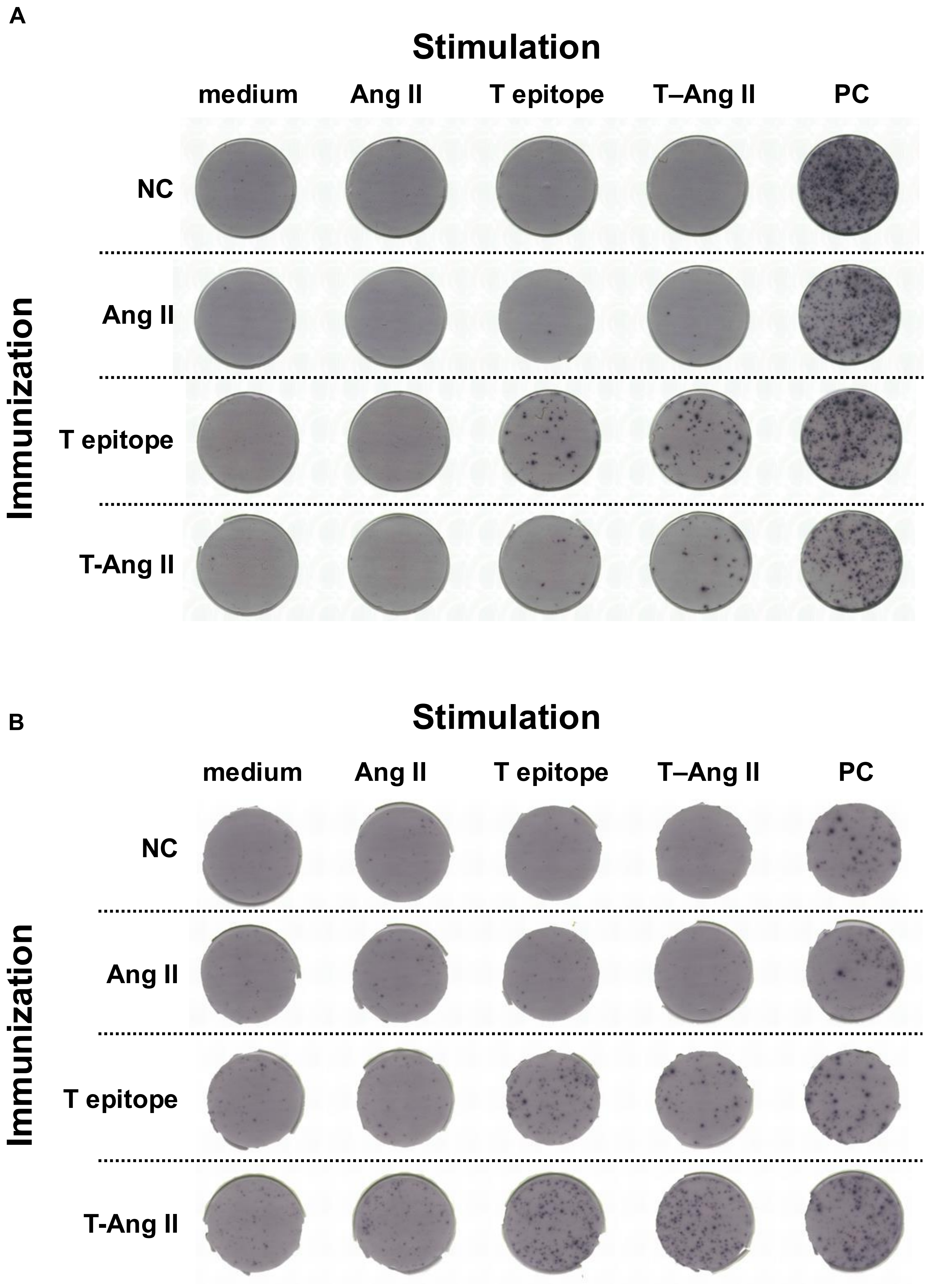

Figure S5. Representative images of ELISpot assay. Related to Figure 3.

Representative images of ELISpot using splenocytes from mice administered peptides three times. IFN-γ (A), IL-4 (B).
